# A clinical trial to compare a 3D‐printed bolus with a conventional bolus with the aim of reducing cardiopulmonary exposure in postmastectomy patients with volumetric modulated arc therapy

**DOI:** 10.1002/cam4.4496

**Published:** 2021-12-23

**Authors:** Yun Zhang, Yuling Huang, Shenggou Ding, Jinghui Liang, Jie Kuang, Qingfeng Mao, Weiliang Ying, Yuxian Shu, Jingao Li, Chunling Jiang

**Affiliations:** ^1^ Department of Radiation Oncology Jiangxi Cancer Hospital of Nanchang University Nanchang PR China; ^2^ School of Public Health Nanchang University Nanchang PR China; ^3^ Key Laboratory of Personalized Diagnosis and Treatment of Nasopharyngeal Carcinoma Nanchang Nanchang PR China; ^4^ Medical College of Nanchang University Nanchang PR China

**Keywords:** 3D‐printed bolus, dosimetry, normal tissue complication probability (NTCP), postmastectomy radiotherapy

## Abstract

**Background:**

We compared the dosimetry, application, and acute toxicity of a 3D‐printed and a conventional bolus for postmastectomy radiotherapy (PMRT) with volumetric modulated arc therapy (VMAT).

Materials and Methods Eligible patients (*n* = 75) with PMRT breast cancer were randomly selected to receive VMAT with a conventional bolus or a 3D‐printed bolus. The primary endpoint was a 10% decrease in the mean heart dose to left‐sided breast cancer patients. The secondary endpoint was a 5% decrease in the mean ipsilateral lung dose to all patients. A comparative analysis was carried out of the dosimetry, normal tissue complication probability (NTCP), acute skin toxicity, and radiation pneumonitis.

**Results:**

Compared to a conventional bolus, the mean heart dose in left‐sided breast cancer was reduced by an average of 0.8 Gy (5.5 ± 1.3 Gy vs. 4.7 ± 0.8 Gy, *p *= 0.035) and the mean dose to the ipsilateral lung was also reduced by an average of 0.8 Gy (12.4 ± 1.0 Gy vs. 11.6 ± 0.8 Gy, *p *< 0.001). The values for V_50Gy_ of the PTV of the chest wall for the 3D‐printed and conventional boluses were 95.4 ± 0.6% and 94.8 ± 0.8% (*p *= 0.026) and the values for the CI of the entire PTV were 0.83 ± 0.02 and 0.80 ± 0.03 (*p *< 0.001), respectively. The NTCP for the 3D‐printed bolus was also reduced to an average of 0.14% (0.32 ± 0.19% vs. 0.18 ± 0.11%, *p *= 0.017) for the heart and 0.45% (3.70 ± 0.67% vs. 3.25 ± 0.18%, *p *< 0.001) for the ipsilateral lung. Grade 2 and Grade 1 radiation pneumonitis were 0.0% versus 7.5% and 14.3% versus 20.0%, respectively (*p *= 0.184).

**Conclusions:**

The 3D‐printed bolus may reduce cardiopulmonary exposure in postmastectomy patients with volumetric modulated arc therapy.

## Graphical abstract

The accuracy of fit of the customized 3D‐printed bolus to the chest wall was improved significantly relative to the conventional bolus for postmastectomy radiotherapy. VMAT plans based on the 3D‐printed bolus resulted in lower dose and normal tissue complication probability to heart and ipsilateral lung than those based on the conventional bolus. The radiation dermatitis and radiation pneumonitis were also reduced on the 3D bolus VMAT plans.

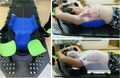

## 1 INTRODUCTION

With advances in treatment technology, survival rates for breast cancer patients are increasing.^1^ Cardiovascular disease has become the leading cause of non‐tumor‐related death in breast cancer patients with long‐term survival,^2^ especially when anthracycline, trastuzumab, and radiotherapy are involved.^3^, ^4^ Protection of cardiopulmonary function is therefore treated as paramount by radiation oncologists. Although there is no clear upper limit for a safe cardiac exposure dose, the mean heart dose (MHD) is an important practical parameter that has been used to predict radiotherapy‐induced heart disease (RIHD) in past decades.^4^, ^5^, ^6^, ^7^

Modified radical surgery continues to play a fundamental role for breast cancer patients in China, and reaching up to 80% of breast cancer patients undergo this surgery in some regions. Radiotherapy for breast cancer after mastectomy contributes to a large degree to a reduction in the risk of local relapse.^6^, ^7^, ^8^ The megavoltage photon beam creates a dose build‐up effect,^9^, ^10^ and to ensure that the dose distribution conforms to the target volume, a bolus is frequently used on the patient's skin.^11^

Recently, several studies have demonstrated that patients benefit from the application of a three‐dimensional (3D) printed bolus.^12^, ^13^, ^14^, ^15^, ^16^, ^17^ However, studies that have suggested a dose reduction for the heart and lung refer only to electron beam radiotherapy for breast cancer patients.^15^, ^16^ Volumetric modulated arc therapy (VMAT) can significantly shorten the length of radiotherapy and improve the efficiency of equipment, and previous research studies have confirmed that VMAT technology can reduce the dose to the heart and ipsilateral lung.^17^, ^18^, ^19^ However, there are no clinical practice data so far on whether VMAT has similar advantages in terms of dosimetry as a 3D‐printed bolus on the chest wall. In this study, we focus on a comparison of the dosimetry characteristics, radiobiology, and acute toxicity of VMAT treatment using a 3D‐printed bolus and a conventional bolus.

### 1.1 MATERIALS AND METHODS Study design and participants

A total of 75 eligible T1–4 and N1–3 breast cancer patients receiving postmastectomy chest wall radiation were recruited between March and December 2020 after approval was granted by the ethics board of our institution. Clinical staging was carried out based on the eighth edition of the TNM classification. The selection criteria included: (i) Karnofsky score ≥80; (ii) an intention to undergo VMAT treatment; and (iii) a requirement for a bolus during treatment. No patient was disenrolled from the clinical trial. Each patient received a series of medical evaluations, including a medical history, a physical examination, a magnetic resonance imaging scan of the chest, blood testing, abdominal ultrasound, chest computed tomography (CT) scan, and a whole‐body bone scan (if necessary) before enrollment.

### 1.2 Randomization and treatment procedures

The random assignment of patients was performed at Nanchang University using a computer‐generated random number code. This was an exploratory open‐label study based on clinical feasibility. There was no formal sample size calculation, as no formal hypothesis test was applied. Depending on clinical feasibility, we planned to include about 70 cases. Patients were randomly distributed in a 1:1 ratio to the 3D‐printed bolus and conventional bolus groups, and the left and right treatment sides formed a stratification factor. All patients understood the treatment assignment, and were fully informed of the available treatment option for bolus. While part of the patients enrolled with 3D printed bolus still opted for conventional bolus due to concerns about the 3D technology, and the appropriate treatment consent forms were signed and kept in the medical record. Thirty‐five patients (19 right‐sided and 16 left‐sided) and 40 patients (20 right‐sided and 20 left‐sided) were enrolled and were assigned either a 3D‐printed bolus or a 5.0‐mm thick conventional bolus.

**FIGURE 1:**
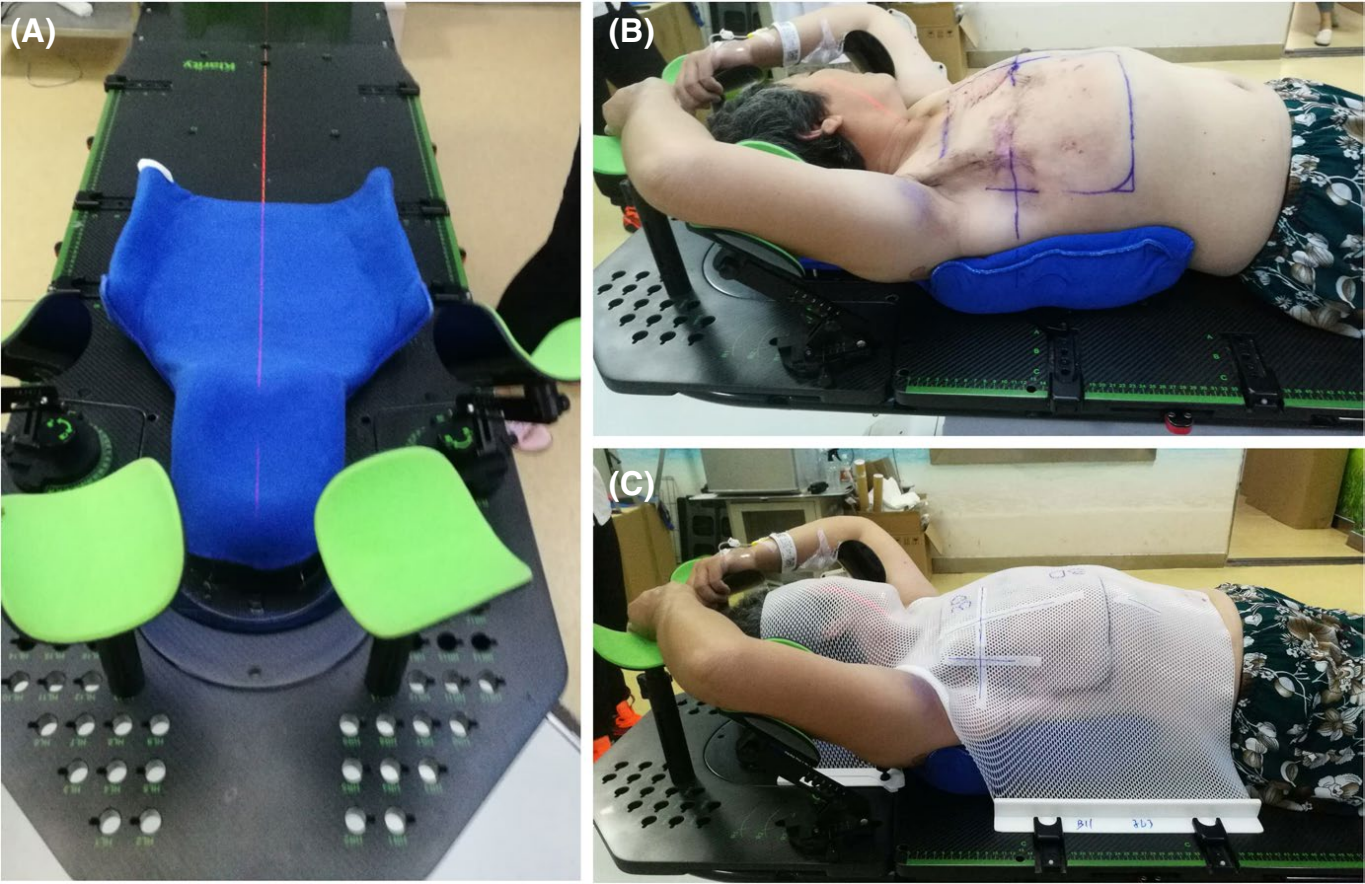
Set up immobilization devices for breast patients (A) shows custom Klarity mold made of a uniform density material to conform to patients’ anatomy and minimize air gaps between the patient and the mold. (B) Shows the patient lying on the mold with her hands on the bracket. (C) Shows a thermoplastic mask affixed to the patient to neck and chest immobilization

All patients were placed in a supine position on a custom mold fixed onto a Solo Align Full Body System (Klarity) with the head tilted to the opposite side, and both upper arms were lifted in abduction. A conventional (standard vinyl gel sheet) bolus or a 3D‐printed bolus was placed on the chest wall as uniformly as possible, to minimize the extent of any air gaps, and tape was applied to assist in conforming to the bolus to the concavities of the patient's surface as necessary. A thermoplastic mask was affixed to the patient to immobilize the neck and chest. The details of setting up the immobilization devices are shown in Figure 1.

The patients were given a CT scan under free breathing with a slice thickness of 5 mm (Siemens Medical Systems). Each clinical target volume (CTV) was delineated according to the breast cancer atlas for radiation therapy planning consensus definitions of the Radiation Therapy Oncology Group (RTOG). Planning target volumes (PTVs) were obtained from each CTV by expanding the 5 mm margin in three dimensions. PTV1 (the ipsilateral chest wall and internal mammary node regions) and PTV2 (the supraclavicular node regions) were restricted to 0 and 4 mm under the skin surface, respectively, and excluded the build‐up region. Organs at risk (OARs) such as the ipsilateral lung, heart, contralateral breast and lung, liver, spinal cord, and trachea were outlined in axial CT sections. CBCT images were designed to verify the positioning error and the geometrical accuracy of the fit of the bolus. Age, tumor classification, and volumes of PTVs for all patients are given in Table 1.

**TABLE 1:** Characteristics of patient for the conventional bolus group and 3D‐printed group

Variable	Conventional bolus group (*n* = 40)	3D‐printed group (*n* = 35)	*p*
Total enrollment, No. (%)	40 (53.33%)	35 (46.67%)	—
Age, mean ± SD, years	47.58 ± 8.06	48.66 ± 9.25	0.590
Tumor stage, No. (%)			
T1	8 (20.00%)	5 (14.29%)	0.858
T2	24 (60.00%)	24 (68.57%)	
T3	6 (15.00%)	4 (14.43%)	
T4	2 (5.00%)	2 (5.71%)	
Node stage, No. (%)			
N1	19 (47.50%)	16 (45.71%)	0.939
N2	12 (30.00%)	9 (25.71%)	
N3	9 (22.50%)	10 (28.57%)	
Volume of PTV1, mean ± SD, cm^3^	380.85 ± 97.12	379.23 ± 86.97	0.940
Volume of PTV2, mean ± SD, cm^3^	173.20 ± 29.48	165.13 ± 35.11	0.283
Volume of PTV, mean ± SD, cm^3^	554.05 ± 113.69	544.36 ± 100.11	0.698

The boluses in this study were designed based on CT images and manufactured using a 3D printer (Rehearsal), involving fused deposition modeling of polylactic acid (PLA) filament and 100% infill.^20^ The design of the bolus for 3D printing was based on a series of CT images with a 1.5 mm slice, and the bolus area was marked with lead wires on the surface of the patient's body according to the extent of surgical clearance during CT scanning. The CT images were then imported into Mimics 19.0 (Materialise, Belgium) to create the 3D skin model. This model was imported into Geomagic Studio 2013 (Geomagi) in STL file format. The skin contours within the target area were extracted with 5 mm, and the thickness of the thin‐walled area was increased appropriately based on cross‐sectional CT images. The individualized 3D printed bolus created in this way was imported into the slicing software for layer‐by‐layer slicing, and the resulting slicing file was input into the 3D printer for printing. The electron density of the 3D‐printed bolus relative to water was set as 1.11 ± 0.01 (range 1.07‒1.13) in our study, which is the same as in previous reports.^12^

For each patient, VMAT plans were created using the Pinnacle treatment planning system (version 9.10) with 6 MV photon energy beams from a Varian TrueBeam linear accelerator. The prescription dose was 50 Gy in 25 fractions, and at least 95% of the PTV received 50 Gy. Dose‐volume histograms (DVHs) were used for a dosimetry analysis. The homogeneity index (HI) of PTV1 and PTV2 was calculated^21^ as $\mathrm{HI}=D_{2\%}‐D_{98\%}/D_{50\%}$. The conformity index (CI) of the PTV as proposed by Paddick et al.^22^ was evaluated as $\mathrm{CI}={\left({\mathrm{VPTV}}_{50}\right)}^{2}/\mathrm{VPTV}\times V_{50}$, where VPTV is the target volume, V_50_ is the volume of the prescribed isodose value, and VPTV_50_ is the volume of the target covered by the prescribed isodose value. The aim, starting objective, and constraints of OARs for two groups planning optimization are specified in Table 2.

**TABLE 2:** The planning parameter, weight, and aims of OARs for VMAT optimization

Structures	Type	Dose (Gy)	Volume (%)	Weight	Aim
Ipsilateral lung	Max DVH	4	38	3	V_5_ Gy ≤50%, V_20_ Gy ≤25% Mean dose ≤8 Gy
	Max DVH	10	28	3	
	Max DVH	18	18	5	
	Max DVH	28	8	3	
	Max DVH	40	1	10	
	Max EUD (*a* = 1)	11	3	3	
Heart for left‐sided	Max DVH	5	15	3	V_30 Gy_ ≤5%, Mean dose ≤8 Gy
	Max DVH	10	8	10	
	Max EUD (*a* = 1)	5	—	5	
Contralateral lung	Max DVH	5	1	1	V_5_ Gy <5% Mean dose ≤3 Gy
	Max EUD (*a* = 1)	1.5	—	3	
Contralateral breast	Max DVH	5	3	1	Mean dose ≤3 Gy
	Max EUD (*a* = 1)	2.0	—	3	
Heart for right‐sided	Max DVH	5	3	1	Mean dose ≤3 Gy
	Max EUD (*a* = 1)	2.0	—	3	
Spinal cord	Max Dose	25	—	10	*D*_max_ ≤30 Gy

### 1.3 Outcomes and follow‐up

MHD is an important practical parameter used to predict radiotherapy‐induced heart disease. In a systematic review of heart doses,^23^ the average MHD was round 8 Gy when the IMC was irradiated in left‐sided breast cancer and 3.3 Gy for right‐sided breast cancer. We previously reported that the MHD was 5.2 ± 0.9 Gy in left breast cancer using a virtual bolus with the VMAT technique.^19^ Since a safe dose for the heart in clinical practice is always controversial, the lower the dose to the heart, the better, in principle. The primary endpoint was therefore a 10% decrease in MHD for left‐sided breast cancer patients, defined as $\begin{array}{c} \left(D_{\mathrm{mean}}\left(3D‐heart\right)‐D_{\mathrm{mean}}\left(conventional‐heart\right)/\right.D_{\mathrm{mean}} \end{array}$ $\begin{array}{c} \left.\left(conventional‐heart\right)\right)\times 100\% \end{array}$. This would mean that the average MHD for left‐sided breast cancer would drop below 5 Gy. The secondary endpoint was a 5% decrease in the mean lung dose (MLD) to the ipsilateral lung, defined as $\begin{array}{c} \left(D_{\mathrm{mean}}\left(3D‐lung\right)‐D_{\mathrm{mean}}\left(conventional‐lung\right)/\right.D_{\mathrm{mean}} \end{array}$ $\begin{array}{c} \left.\left(conventional‐lung\right)\right)\times 100\% \end{array}$. The normal tissue complication probability (NTCP) for radiation‐induced pneumonitis and mortality was computed for the lung and heart using the Lyman‒Kutcher‒Berman^24^ and relative seriality models, respectively.^25^ Toxicity was scored using the Radiation Therapy Oncology Group (RTOG) radiation morbidity scoring system.^26^ No data on long‐term toxicity were collected. Radiation pneumonitis toxicity was evaluated within 12 weeks after radiotherapy.

### 1.4 Statistical analysis

An independent two‐sample *t*‐test was used to compare the air gaps between the bolus and skin and the dosimetry parameters. A chi‐squared test was used for a comparison of radiation toxicity. Values of *p *≤ 0.05 were considered statistically significant. All statistical analyses were two‐sided and were performed using SPSS version 18.0 (IBM Corporation).

## 2 RESULTS

**FIGURE 2:**
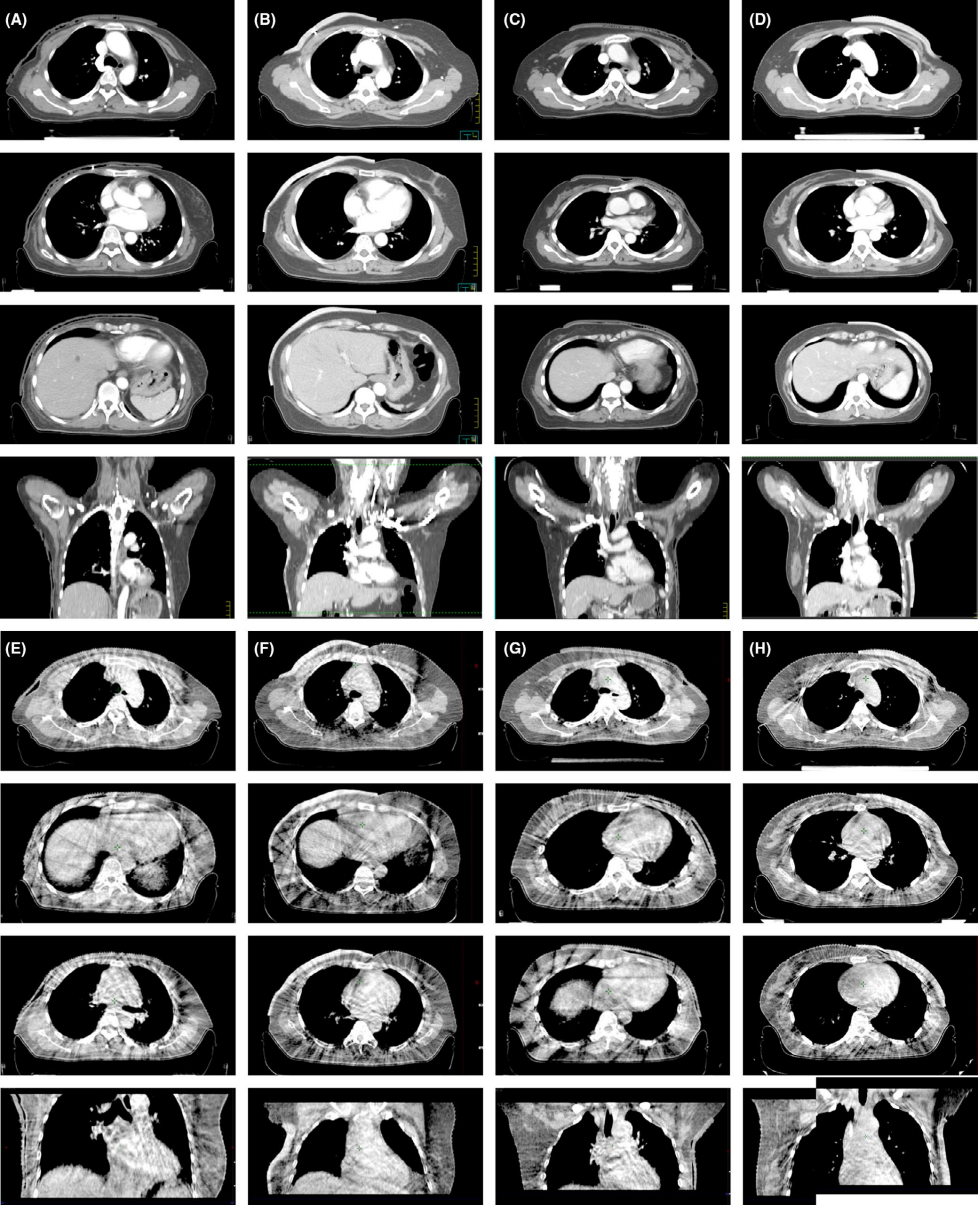
Examples of conventional bolus and 3D‐printed bolus CT (A–D) and CBCT images (E–H). A and E were right‐sided breast with the conventional bolus, B and F were right‐sided breast with the 3D‐printed bolus, C and G were left‐sided breast with the conventional bolus, and D and H were left‐sided breast with 3D printed bolus

**FIGURE 3:**
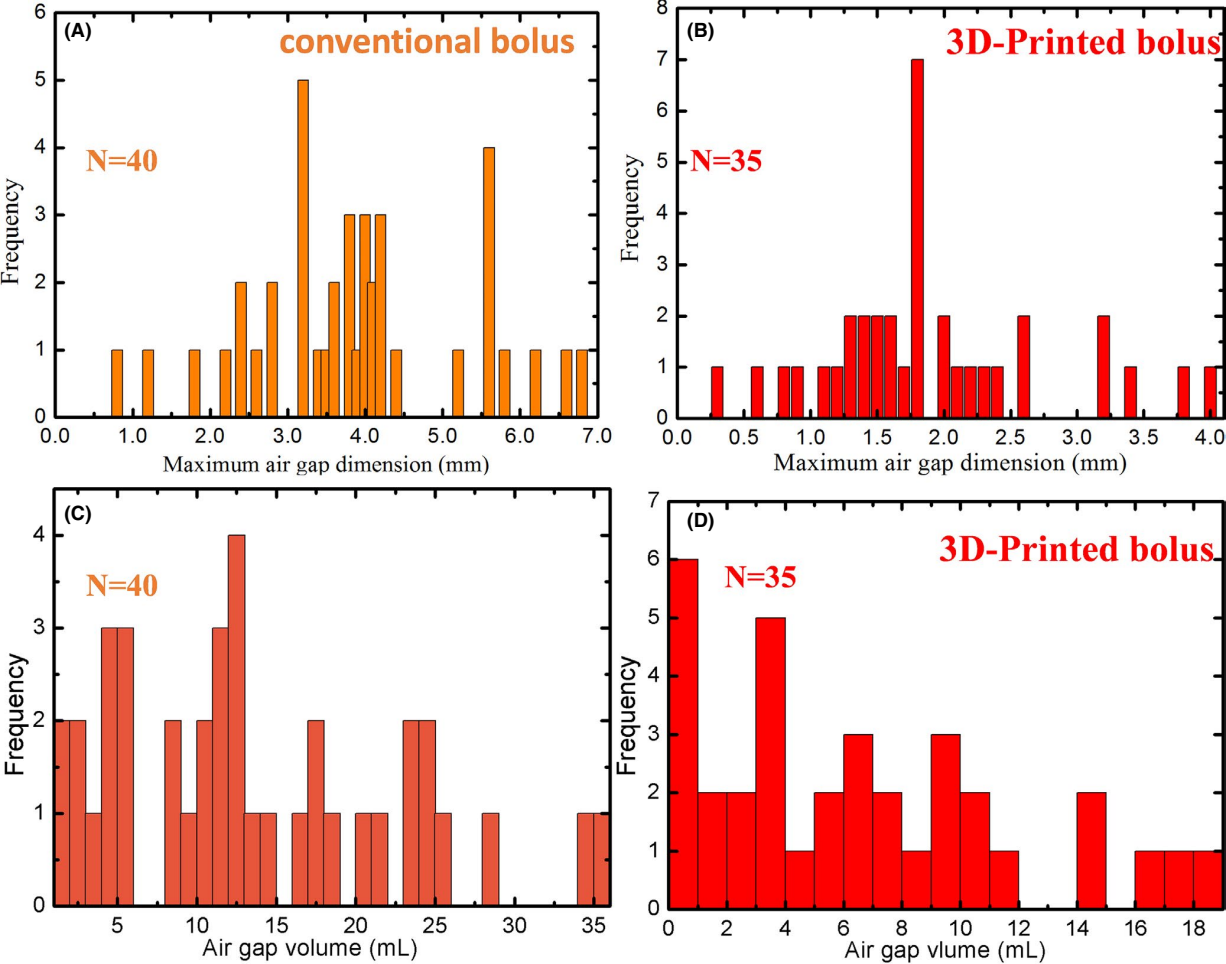
Frequency histogram of the maximum dimension and volume of the air gap between bolus and patient surface as measured on CT images (A, C) conventional bolus (B, D) and 3D‐printed bolus

Figure 2 shows CT and CBCT images of four patients with a conventional bolus (images A, C, E, and G) and a 3D‐printed bolus (images B, D, F, and H), respectively. The 3D‐printed bolus was superior, as smaller air gaps between the bolus and skin can be seen from the images. A frequency histogram for the maximum dimension of the air gap in the CT measurements is given in Figure 3. These distributions have different frequencies, where the frequency is 3.2 mm for the conventional bolus and 1.8 mm for the 3D‐printed bolus. For all patients, the maximum mean air gap was 3.9 ± 1.4 mm for the conventional bolus and only 1.9 ± 0.9 mm for the 3D‐printed bolus (*p *< 0.001). In addition, the air gap volume differed significantly (*p *< 0.001), with the 3D printed bolus resulting in lower values. Over all observations, the mean total air gap volumes were 6.5 ± 5.1 and 14.1 ± 8.8 ml for 3D printed bolus and for conventional bolus, respectively. These larger air gaps occurred at regions of surface complexity and, in particular, locations featuring large convexities or concavities (see Figure 2).

**FIGURE 4:**
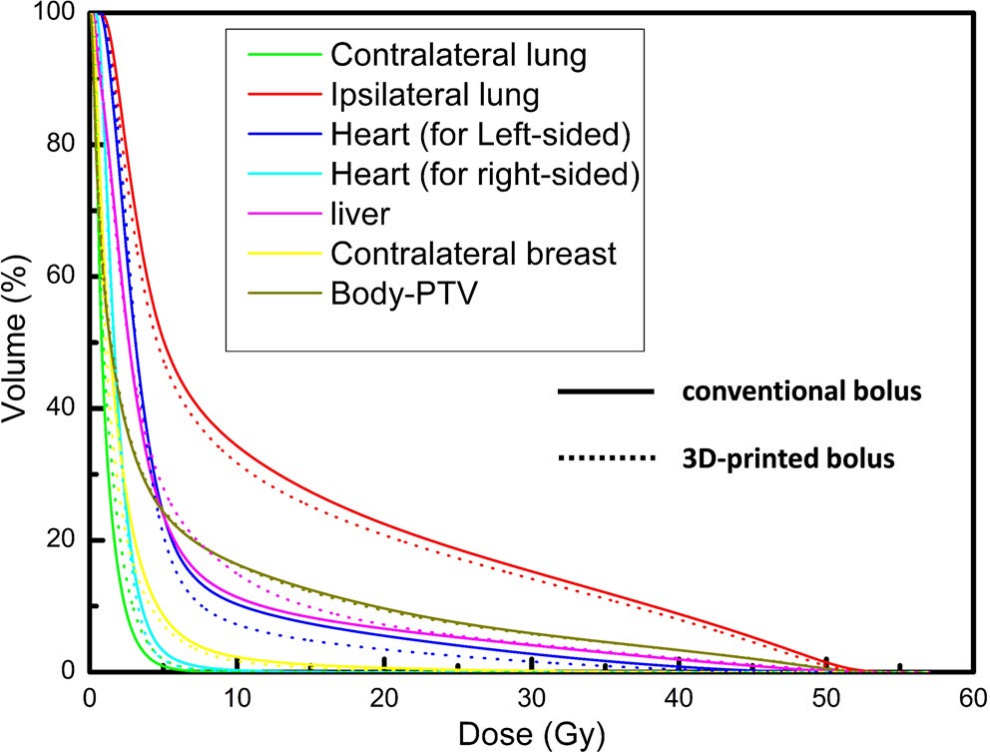
Average dose‐volume histogram (DVH) comparison for OARs with conventional bolus (solid line) and 3D printed bolus (dot line)

**FIGURE 5:**
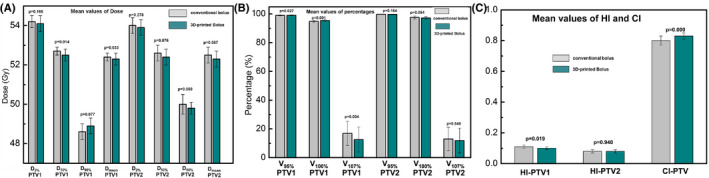
PTV dosimetry parameters for conventional and 3D‐printed bolus with VMAT plans. (A) Mean values of dose. (B) Mean values of percentage. (C) Mean values of HI and CI

Figure 4 shows average dose‐volume histograms (DVHs) for OARs with all conventional bolus plans and all 3D‐printed bolus plans. The relevant dosimetry parameters for the OARs (ipsilateral lung, heart, contralateral lung and breast, liver, and all normal tissue [Body‐PTV]) are presented in Table 3. Compared to the conventional bolus, the MHD was reduced by an average of 14.55% (0.8 Gy) for the left‐sided breast, while the V20 and mean dose for the ipsilateral lung were decreased by an average of 7.56% (1.7%) and 6.45% (0.8 Gy), respectively. No substantial differences were observed for the other OARs. PTV dosimetry parameters for the conventional and 3D‐printed bolus plans for all patients are presented in Figure 5. For PTV1, the coverage of the 100% prescription dose was higher, with a score of 95.4 ± 0.6% versus 94.8 ± 0.8% (*p *= 0.001), and the HI in the 3D‐printed bolus was better than for the conventional bolus (0.10 ± 0.01 vs. 0.11 ± 0.01, *p *= 0.019). No substantial differences were found for PTV2. For the overall PTV, the mean CI for the 3D‐printed bolus was higher than for the conventional bolus (0.83 ± 0.02 vs. 0.80 ± 0.03, *p* < 0.001).

**TABLE 3:** Dosimetric difference between conventional bolus and 3D printed bolus on OARs

Structure	Parameters	Conventional bolus	3D‐printed bolus	Difference	*p*
Ipsilateral lung	V_5_ (%)	50.4 ± 4.2	47.3 ± 3.3	−3.1	<0.001
	V_10_ (%)	34.3 ± 2.9	31.7 ± 2.2	−2.6	<0.001
	V_20_ (%)	22.5 ± 2.3	20.8 ± 1.8	−1.7	<0.001
	V_30_ (%)	15.3 ± 1.9	14.2 ± 1.5	−1.1	0.005
	V_40_ (%)	8.8 ± 1.7	8.0 ± 1.1	−0.8	0.013
	MLD (Gy)	12.4 ± 1.0	11.6 ± 0.8	−0.8	<0.001
Heart (left‐sided)	V_5_ (%)	24.0 ± 8.8	20.8 ± 7.1	−3.2	0.252
	V_10_ (%)	10.3 ± 3.7	7.2 ± 3.1	−3.1	0.011
	V_20_ (%)	5.5 ± 2.1	3.4 ± 1.6	−2.1	0.002
	V_30_ (%)	2.8 ± 1.3	1.6 ± 0.9	−1.2	0.004
	V_40_ (%)	0.8 ± 0.5	0.4 ± 0.3	−0.4	0.007
	MHD (Gy)	5.5 ± 1.3	4.7 ± 0.8	−0.8	0.035
Heart (right‐sided)	MHD (Gy)	2.0 ± 0.4	2.0 ± 0.3	−0.0	0.487
Contralateral lung breast	*D*_mean_ (Gy)	2.1 ± 0.4	1.9 ± 0.6	−0.2	0.183
Contralateral breast lung	MLD (Gy)	1.2 ± 0.2	1.2 ± 0.3	0.0	0.506
Liver (right‐sided)	V_5_ (%)	23.8 ± 11.6	27.9 ± 17.3	4.1	0.385
	V_10_ (%)	11.4 ± 5.2	15.0 ± 11.7	3.6	0.223
	V_20_ (%)	6.6 ± 3.1	7.2 ± 3.9	0.6	0.592
	V_30_ (%)	4.1 ± 2.1	4.3 ± 2.2	0.2	0.788
	V_40_ (%)	1.9 ± 1.3	2.1 ± 1.3	0.2	0.661
	*D*_mean_ (Gy)	5.4 ± 1.9	5.6 ± 2.5	0.2	0.810
Body‐PTV	V_5_ (%)	24.3 ± 2.4	24.6 ± 2.4	0.3	0.596
	V_10_ (%)	16.4 ± 1.7	16.2 ± 1.5	−0.2	0.746
	V_20_ (%)	9.7 ± 0.9	9.3 ± 1.0	−0.4	0.150
	V_30_ (%)	5.9 ± 0.7	5.8 ± 0.7	−0.1	0.437
	V_40_ (%)	3.3 ± 0.4	3.3 ± 0.5	−0.0	0.870
	*D*_mean_ (Gy)	5.8 ± 0.5	5.7 ± 0.5	−0.1	0.336

Compared with the conventional bolus, lower values for the NTCP of the heart and ipsilateral lung were also found for the 3D‐printed bolus; these were reduced by an average of 0.14% (0.32 ± 0.19% vs. 0.18 ± 0.11%, *p *= 0.017) and 0.45% (3.70 ± 0.67% vs. 3.25 ± 0.18%, *p *= 0.001). No ≥Grade 2 radiation pneumonitis (RP) occurred with the 3D‐printed bolus, whereas three patients from the conventional bolus group were identified to have ≥Grade 2 RP. With or without a 3D‐printed bolus, Grade 1 RP occurred in 14.3% versus 20.0% of the cases (*X* ^2^ = 3.39, *p *= 0.184). Twenty‐nine (38.7%) patients in total were identified as having ≥Grade 2 radiation dermatitis (RD) which occurred where the bolus was applied, and the difference in the occurrence rates of ≥Grade 2 RD between the conventional bolus (17/40) and the 3D‐printed bolus (12/35) was not statistically significant (*X* ^2 ^= 0.531, *p *= 0.466).

## 3 DISCUSSION

This study aimed to show that the use of an individualized 3D‐printed bolus combined with VMAT technology can reduce the irradiated dose to heart and lung in PMRT, resulting in a reduced incidence of acute toxicity. Our results revealed a 14.5% (0.8 Gy) decrease in the MHD for left‐sided breast cancer treated with a 3D‐printed bolus, compared to a conventional standard super‐flat bolus. Our results are similar to those of previous studies on electronic electron beam technology for breast cancer patients.^15^, ^16^

In an earlier evaluation of clinical radiation treatment planning, the recommended dose limit for the heart was set at V_25_ <10%. However, Beaton found that even if V_25_ did not exceed 10% in all patients with left‐sided breast cancer, the 10‐year incidence of cardiovascular disease was still 1.7%.^27^ He therefore suggested that the MHD should be used as a criterion for considering cardiac limits. The left anterior descending (LAD) artery is the branch of the coronary artery that is most likely to be damaged by radiotherapy, so some scholars have also proposed using the mean dose of the LAD to predict the incidence of major coronary events, although Shah found that this index was less predictive than MHD.^28^ Darby proposed that the incidence of major coronary events increased linearly with increasing MHD, with a 7.4% increase per gray level.^4^ In other words, compared to a conventional bolus, left‐sided breast cancer patients treated with a 3D‐printed bolus may be reduced by 5.92% in MHD. By looking at a meta‐analysis of data from 75 trials in which patients were randomly assigned to breast cancer radiotherapy versus no radiotherapy, Taylor found a cardiac mortality ratio of 1.3 per 1 Gy of MHD.^29^ Although Van believed that MHD alone might be obsolete as a risk assessment index for cardiac injury, and proposed a new index based on the volume of the left ventricle receiving 5 Gy as a predictor for an acute coronary event,^30^ this index has not been widely used in clinical practice. Trott suggested that NTCP based on anatomical dose distribution can also be predictive of cardiopulmonary radiation risk.^31^ In this study, the results for the 3D‐printed bolus showed a lower NTCP on the heart (0.14%). For right‐sided breast cancer, although the dose to the heart did not change between the 3D‐printed bolus and the conventional bolus, this value was lower than the results reported in the literature.^23^

In addition to the lower dose to the heart, the dose to the ipsilateral lung was also reduced (6.45%, 0.8 Gy), which resulted in a decrease in NTCP. Although patients with a 3D‐printed bolus had a lower incidence of Grade 1 and 2 RP, the difference in the outcomes between the two groups was not statistically significant. The frequency of symptomatic RP has been reported as 1%–7% after treating local and regional nodes with a total dose of 45–50 Gy.^32^, ^33^ We reduced the volume of irradiation dose to 5–30 Gy in the ipsilateral lung using the VMAT technique, especially in the 3D‐printed bolus. In the current study, no patients in the 3D‐printed bolus group developed Grade 2 RP. Furthermore, for clinical postmastectomy plans, the dose distribution for the PTV of the chest wall with a 3D‐printed bolus did not suffer from the problems experienced by the conventional bolus. In our study, the PTV of the chest wall for the V_100%_ range was 91.7%–95.6% for the conventional bolus and 94.5%–97.8% for the 3D‐printed bolus. The results were also considerably more homogeneous than for a conventional bolus, and this improvement in the homogeneity of irradiation will reduce the rates of acute complications and long‐term fibrosis.^4^ Our results are inconsistent with previous results for the electronic irradiation of the chest wall,^34^ which had a similar target dose coverage.

The 3D‐printed bolus reduces the dose to the heart and lung, and provides higher coverage of the chest wall, mainly because it gives a better fit and the bolus is thicker on a thinner chest wall. In terms of the accuracy of fit between the skin and the bolus, the 3D‐printed bolus was superior to the conventional bolus as its outline was more consistent with the chest wall (Figure 2). It is encouraging to see that the average dimension of the maximum air gap for both the conventional and the 3D‐printed bolus was lower than previously reported,^7^ and there were no larger air gaps (>10 mm) for either bolus. This could be ascribed to the use of a thermoplastic mold for immobilization. The radiotherapist pressed the thermoplastic mold above the bolus to improve the fit between the bolus and skin, and continued to press as the mold cooled. The average MHD and MLD using a 5‐mm virtual bolus were 5.2 and 11.5 Gy, respectively, in our previous study,^19^ which were lower than the values for the real conventional bolus of 5.5 and 12.4 Gy. This further demonstrates that air gaps increase the exposure dose to the heart and ipsilateral lungs.

In addition to the effect of the air gap on the dose distribution of the OAR and targets, the thickness of the bolus will also change the dose to the skin.^35^, ^37^ Monajemi et al. showed that average skin doses in postmastectomy VMAT treatments with a 3, 5, and 10 mm bolus were 0.96, 1.03, and 1.04, respectively.^32^ It should be noted that the thickness of the chest wall after mastectomy is uneven. The differences in the chest wall thickness for the majority of the patients who took part in this study were between 6 and 8 mm, and the largest exceeded 14 mm. It is therefore not appropriate to use boluses of uniform thickness. The results of previous studies^36^ confirm that for a 3D conformal electron beam therapy of the chest wall after radical mastectomy, modifications to the patient's skin surface with a variable bolus can improve the coverage and homogeneity of the target dose. This method is also suitable for x‐rays. Yoon et al. showed^37^ that the dose reduction due to the bolus was more significant at shorter distances from the beam, and most of the dose reductions occurred in the first 2 cm of depth and stopped at a 4 cm of depth for IMRT or VMAT. We therefore designed the thickness of the bolus based on the inner profile of the patient's chest wall, so that the outline of the bolus would be similar to that of the chest wall, thus ensuring that there was sufficient bolus over the thinner chest wall for dose build‐up. The average MHD using a 5 mm virtual bolus in our previous report^19^ was also lower than for the 3D‐printed bolus (5.2 vs. 4.7 Gy), while the MLD was lower for the virtual bolus (115 vs. 11.6 Gy). It may be that the effect of the air gap is greater than the effect of the thickness of the bolus.

To further confirm that the air gap and the thickness of the bolus can modify the dose to the heart and ipsilateral lung, three patients with left‐sided breast cancer for conventional bolus who had a high heart dose were selected. We modified the bolus by filling the air gap with virtual water material and adding the 5 mm thickness of the virtual bolus to the conventional bolus, and then re‐optimizing it based on the treatment planning system. The new results showed that the MLD was reduced by 0.6, 1.2, and 0.7 Gy, respectively, and the ipsilateral MLD was reduced by 1.0, 0.9, and 0.9 Gy, respectively. More specifically, the coverage of the prescription dose for the PTV of the chest wall was increased by 0.1%, 2.5%, and 0.4%, respectively. The conformity index was increased by 0.04, 0.03, and 0.06, respectively, and there were no significant changes in the other OARs. These results mean that the completely fitted bolus and the thicker bolus over a thin chest wall can reduce the radiation dose to the heart and lung, and improve the target dose coverage of normal tissue on these areas. A custom 3D‐printed bolus was therefore more suitable for radiotherapy following mastectomy, and also offered clinical benefits due to the improvement in PTV dosimetry and the decrease in the OAR dosimetry.

In this study, we found that there was no difference in the radiation dose to the liver between the conventional bolus and 3D‐printed bolus for the right‐sided breast. This is likely to be because only a small part of the liver was located behind the PTV of the chest wall, meaning that it had little effect on the overall radiation dose to the liver.

The RP was decreased for the 3D‐printed bolus compared to the conventional bolus, but the difference was not statistically significant, which may have been due to the small sample used and the absolute value of differences in mean the ipsilateral lung between 3D‐printed bolus and conventional bolus was small in this case. In addition, the probability of RP is relatively low for breast cancer, and it may not only be related to the dose of ipsilateral lung. The RD ≥Grade 2 (38.7%) was lower than that reported in other studies.^38^, ^39^, ^40^ As in previous reports, the frequency of ≥Grade 2 RD was 40%–90% for patients treated with a conventional technique,^35^ and 42% for patients treated with IMRT.^39^ The results of Arsenault's study (38%) are similar to our findings.^40^ Chen et al.^41^ found that the risk factor for acute skin toxicity in adjuvant breast radiotherapy was mainly related to V_107%_ and V_110%_. This corresponds to the dosimetry results of this study.

There are several potential limitations of this study that merit consideration. The primary limitations are the small sample size and the fact that fewer left‐sided patients received the 3D‐printed bolus. A further limitation relates to missing data for long‐term clinical follow‐up. Additional studies are therefore required to examine these issues.

## 4 CONCLUSIONS

The use of a 3D‐printed bolus in VMAT plans achieved better dose coverage and homogeneity in the PTV of the chest wall and the ipsilateral lung/heart irradiated dose, which resulted in reductions in the NTCP for those OARs and the rates of RD and RP. In conclusion, applications of new technologies such as 3D printing may enable more patients to reap the benefits of adjuvant radiation therapy for breast cancer treatment.

## CONFLICT OF INTEREST

None.

## AUTHOR CONTRIBUTIONS

Yun Zhang: Data curation, methodology, project administration, writing – original draft, and writing – review and editing. Yuling Huang: Data curation, formal analysis, and investigation. Shenggou Ding: Investigation and project administration. Jinghui Liang: Data curation and investigation. Jie Kuang: Formal analysis. Qingfeng Mao: Investigation. Weiliang Ying: Methodology. Yuxian Shu: Investigation. Jingao Li: Writing, review, and editing. Chunling Jiang: Conceptualization, formal analysis, funding acquisition, writing – review, and editing.

## Clinical trial registration number

This study also has been registered in the ClinicalTrials.gov. The ID is NCT04685460.

## ETHICAL APPROVAL STATEMENT

The study has been approved by the Institution Review Board and Ethics Committee of Jiangxi Cancer Hospital of Nanchang University, and the approval number is 2020ky025.

## Data Availability

The data generated or analyzed during the current study are available from the corresponding author upon reasonable request.

## References

[1] Siegel RL , Miller KD , Jemal A . Cancer statistics, 2017. Ca A Cancer J Clin. 2017;67(1):7‐30. 10.3322/caac.21387 28055103

[2] Bradshaw PT , Stevens J , Khankari N , et al. Cardiovascular disease mortality among breast cancer survivors. Epidemiology. 2016;27(1):6‐13. 26414938 10.1097/EDE.0000000000000394 PMC4666721

[3] Henson KE , McGale P , Darby SC . Cardiac mortality after radiotherapy, chemotherapy and endocrine therapy for breast cancer: cohort study of 2 million women from 57 cancer registries in 22 countries. Int J Cancer. 2020;147(5):1437‐1449. 32022260 10.1002/ijc.32908 PMC7496256

[4] Darby SC , Ewertz M , Mcgale P , et al. Risk of ischemic heart disease in women after radiotherapy for breast cancer. N Engl J Med. 2013;368(11):987‐998. 23484825 10.1056/NEJMoa1209825

[5] Jacob S , Camilleri J , Derreumaux S , et al. Is mean heart dose a relevant surrogate parameter of left ventricle and coronary arteries exposure during breast cancer radiotherapy: a dosimetric evaluation based on individually‐determined radiation dose (BACCARATstudy). Radiat Oncol. 2019;14(1):29. 30732640 10.1186/s13014-019-1234-z PMC6367844

[6] Overgaard M , Hansen PS , Overgaard J , et al. Postoperative radiotherapy in high‐risk premenopausal women with breast cancer who receive adjuvant chemotherapy. Danish Breast Cancer Cooperative Group 82b Trial. N Engl J Med. 1997;337:949‐955. 9395428 10.1056/NEJM199710023371401

[7] Ragaz J , Jackson SM , Le N , et al. Adjuvant radiotherapy and chemotherapy in node‐positive premenopausal women with breast cancer. N Engl J Med. 1997;337:956‐962. 9309100 10.1056/NEJM199710023371402

[8] Overgaard M , Nielsen HM , Overgaard J . Is the benefit of postmastectomy irradiation limited to patients with four or more positive nodes, as recommended in international consensus reports? A subgroup analysis of the DBCG 82 b&c randomized trials. Radiother Oncol. 2007;82:247‐253. 17306393 10.1016/j.radonc.2007.02.001

[9] Loevinger R . A formalism for calculation of absorbed dose to a medium from photon and electron beams. Med Phys. 1981;8(1):1‐12. 7207415 10.1118/1.594901

[10] Mihaylov IB , Penagaricano J , Moros EG . Quantification of the skin sparing effect achievable with high‐energy photon beams when carbon fiber tables are used. Radiother Oncol. 2009;93(1):147‐152. 19515440 10.1016/j.radonc.2009.05.008

[11] Hsu SH , Roberson PL , Chen Y , et al. Assessment of skin dose for breast chest wall radiotherapy as a function of bolus material. Phys Med Biol. 2008;53(10):2593‐2606. 18441412 10.1088/0031-9155/53/10/010

[12] Robar JL , Moran K , Allan J , et al. Intrapatient study comparing 3D‐printed bolus versus standard vinyl gel sheet bolus for postmastectomy chest wall radiation therapy. Pract Radiat Oncol. 2018;8(4):221‐229. 29452866 10.1016/j.prro.2017.12.008

[13] Park S‐Y , Choi CH , Park JM , et al. A patient‐specific polylactic acid bolus made by a 3D printer for breast cancer radiation therapy. PLoS One. 2016;11(12):e0168063. 27930717 10.1371/journal.pone.0168063 PMC5145239

[14] Park K , Park S , Jeon M‐J , et al. Clinical application of 3D‐printed‐step‐bolus in post‐total‐mastectomy electron conformal therapy. Oncotarget. 2017;8(15):25660‐25668. 27784001 10.18632/oncotarget.12829 PMC5421959

[15] Li N , Tian Y , Jin J , et al. A customized tissue compensator with 3‐dimensional print technique for chest wall electron irradiation. Int J Radiat Oncol Biol Phys. 2016;96(2):E636. doi: 10.1016/j.ijrobp.2016.06.2221

[16] Yang K , Park W , Ju SG , et al. Heart‐sparing radiotherapy with three‐dimensional printing technology after mastectomy for patients with left breast cancer. Breast J. 2019;25(4):682‐686. 31077484 10.1111/tbj.13304

[17] Popescu CC , Olivotto IA , Beckham WA , et al. Volumetric modulated arc therapy improves dosimetry and reduces treatment time compared to conventional intensity‐modulated radiotherapy for locoregional radiotherapy of left‐sided breast cancer and internal mammary nodes. Int J Radiat Oncol Biol Phys. 2010;76:287‐295. 19775832 10.1016/j.ijrobp.2009.05.038

[18] Hu J , Han G , Lei YU , et al. Dosimetric comparison of three radiotherapy techniques in irradiation of left‐sided breast cancer patients after radical mastectomy. Biomed Res Int. 2020;2020. doi: 10.1155/2020/7131590 PMC7085359 32258140

[19] Zhang Y , Huang Y , Ding S , et al. A dosimetric and radiobiological evaluation of VMAT following mastectomy for patients with left‐sided breast cancer. Radiat Oncol. 2021;16(1):171. 34488817 10.1186/s13014-021-01895-2 PMC8422660

[20] Zhao Y , Moran K , Yewondwossen M , et al. Clinical applications of 3‐dimensional printing in radiation therapy. Med Dosim. 2017;42:150‐155. 28495033 10.1016/j.meddos.2017.03.001

[21] Hodapp N . The ICRU Report 83: prescribing, recording and reporting photon‐beam intensity‐modulated radiation therapy (IMRT). Strahlenther Onkol. 2012;188(1):97‐99. 22234506 10.1007/s00066-011-0015-x

[22] Paddick I . A simple scoring ratio to index the conformity of radiosurgical treatment plans. Technical note. J Neurosurgery. 2000;93(Suppl 3):219‐222. 10.3171/jns.2000.93.supplement 11143252

[23] Taylor CW , Wang Z , Macaulay E , Jagsi R , Duane F , Darby SC . Exposure of the heart in breast cancer radiation therapy: a systematic review of heart doses published during 2003 to 2013. Int J Radiat Oncol Biol Phys. 2015;93(4):845‐853. 26530753 10.1016/j.ijrobp.2015.07.2292

[24] Seppenwoolde Y , Lebesque JV , de Jaeger K , et al. Comparing different NTCP models that predict the incidence of radiation pneumonitis. Normal tissue complication probability. Int J Radiat Oncol Biol Phys. 2003;55(3):724‐735. 12573760 10.1016/s0360-3016(02)03986-x

[25] Gagliardi G , Lax I , Ottolenghi A , Rutqvist LE . Long‐term cardiac mortality after radiotherapy of breast cancer–application of the relative seriality model. Br J Radiol. 1996;69(825):839‐846. 8983588 10.1259/0007-1285-69-825-839

[26] Radiation Therapy Oncology Group . Acute radiation morbidity scoring criteria. Accessed 2 October 2017. http://www.rtog.org

[27] Beaton L , Bergman A , Nichol A , et al. Cardiac death after breast radiotherapy and the QUANTEC cardiac guidelines. Clin Transl Radiat Oncol. 2019;19:39‐45. 31485490 10.1016/j.ctro.2019.08.001 PMC6715791

[28] Shah C , Badiyan S , Berry S , et al. Cardiac dose sparing and avoidance techniques in breast cancer radiotherapy. Radiother Oncol. 2014;112(1):9‐16. 24813095 10.1016/j.radonc.2014.04.009

[29] Taylor C , Correa C , Duane FK , et al. Estimating the risks of breast cancer radiotherapy: evidence from modern radiation doses to the lungs and heart and from previous randomized trials. J Clin Oncol. 2017;35(15):1641‐1649. 28319436 10.1200/JCO.2016.72.0722 PMC5548226

[30] Van den Bogaard VA , Ta BD , van der Schaaf A , et al. Validation and modification of a prediction model for acute cardiac events in patients with breast cancer treated with radiotherapy based on three‐dimensional dose distributions to cardiac substructures. J Clin Oncol. 2017;35(11):1171‐1178. 28095159 10.1200/JCO.2016.69.8480 PMC5455600

[31] Trott K‐R , Doerr W , Facoetti A , et al. Biological mechanisms of normal tissue damage: importance for the design of NTCP models. Radiother Oncol. 2012;105(1):79‐85. 22748390 10.1016/j.radonc.2012.05.008

[32] Kahan Z , Csenki M , Varga Z , et al. The risk of early and late lung sequelae after conformal radiotherapy in breast cancer patients. Int J Radiat Oncol Biol Phys. 2007;68(3):673‐681. 17350177 10.1016/j.ijrobp.2006.12.016

[33] Matzinger O , Heimsoth I , Poortmans P , et al. Toxicity at three years with and without irradiation of the internal mammary and medial supraclavicular lymph node chain in stage I to III breast cancer (EORTC trial 22922/10925). Acta Oncol. 2010;49(1):24‐34. 20100142 10.3109/02841860903352959

[34] Li G , Kuo L , Kowalski A , et al. Clinical evaluation of soft 3D‐printed bolus in radiotherapy of nasal cancer. Int J Radiat Oncol Biol Phys. 2019;105(1):E686. doi: 10.1016/j.ijrobp.2019.06.916

[35] Monajemi TT , Oliver PAK , Day A , et al. In search of a one plan solution for VMAT post‐mastectomy chest wall irradiation. J Appl Clin Med Phys. 2020;21:216‐223. 10.1002/acm2.12948 PMC7484836 32592451

[36] Perkins GH , McNeese MD , Antolak JA , et al. A custom three‐dimensional electron bolus technique for optimization of postmastectomy irradiation. Int J Radiat Oncol Biol Phys. 2001;51(4):1142‐1151. 11704339 10.1016/s0360-3016(01)01744-8

[37] Yoon J , Xie Y , Zhang R . Evaluation of surface and shallow depth dose reductions using a Superflab bolus during conventional and advanced external beam radiotherapy. J Appl Clin Med Phys. 2018;19(2):138‐143. 29427312 10.1002/acm2.12269 PMC5849823

[38] Boström Å , Lindman H , Swartling C , et al. Potent corticosteroid cream (mometasone furoate) significantly reduces acute radiation dermatitis: results from a double‐blind, randomized study. Radiother Oncol. 2001;59(3):257‐265. 11369066 10.1016/s0167-8140(01)00327-9

[39] Ma J , Li J , Xie J , et al. Post mastectomy linac IMRT irradiation of chest wall and regional nodes: dosimetry data and acute toxicities. Radiat Oncol. 2013:8:81. 23566488 10.1186/1748-717X-8-81 PMC3643842

[40] Arsenault J , Parpia S , Goldberg M , et al. Acute toxicity and quality of life of hypofractionated radiation therapy for breast cancer. Int J Radiat Oncol Biol Phys. 2020;107(5):943‐948. 32334033 10.1016/j.ijrobp.2020.03.049

[41] Chen MF , Chen WC , Lai CH , Hung CH , Liu KC , Cheng YH . Predictive factors of radiation‐induced skin toxicity in breast cancer patients. BMC Cancer. 2010;23(10):508. 10.1186/1471-2407-10-508 PMC2955039 20860847

